# The promotion action of AURKA on post-ischemic angiogenesis in diabetes-related limb ischemia

**DOI:** 10.1186/s10020-023-00635-4

**Published:** 2023-03-28

**Authors:** Tao Bai, Mingxing Li, Yuanfeng Liu, Zhentao Qiao, Xusheng Zhang, Yafeng Wang, Zhiwei Wang

**Affiliations:** 1 Department of Vascular and Endovascular Surgery, The First Affiliated Hospital of Zhengzhou University, 1 East Jianshe Road, Zhengzhou, People’s Republic of China; 2 Department of Vascular Surgery, Gongyi City People’s Hospital, Gongyi, People’s Republic of China

**Keywords:** Diabetic limb ischemia, AURKA, Angiogenesis

## Abstract

**Background:**

Diabetes-related limb ischemia is a challenge for lower extremity amputation. Aurora Kinase A (AURKA) is an essential serine/threonine kinase for mitosis, while its role in limb ischemia remains unclear.

**Method:**

Human microvascular endothelial cells (HMEC-1) were cultured in high glucose (HG, 25 mmol/L d-glucose) and no additional growth factors (ND) medium to mimic diabetes and low growth factors deprivation as in vitro model. Diabetic C57BL/6 mice were induced by streptozotocin (STZ) administration. After seven days, ischemia was surgically performed by left unilateral femoral artery ligation on diabetic mice. The vector of adenovirus was utilized to overexpress AURKA in vitro and in vivo.

**Results:**

In our study, HG and ND-mediated downregulation of AURKA impaired the cell cycle progression, proliferation, migration, and tube formation ability of HMEC-1, which were rescued by overexpressed AURKA. Increased expression of vascular endothelial growth factor A (VEGFA) induced by overexpressed AURKA were likely regulatory molecules that coordinate these events. Mice with AURKA overexpression exhibited improved angiogenesis in response to VEGF in Matrigel plug assay, with increased capillary density and hemoglobin content. In diabetic limb ischemia mice, AURKA overexpression rescued blood perfusion and motor deficits, accompanied by the recovery of gastrocnemius muscles observed by H&E staining and positive Desmin staining. Moreover, AURKA overexpression rescued diabetes-related impairment of angiogenesis, arteriogenesis, and functional recovery in the ischemic limb. Signal pathway results revealed that VEGFR2/PI3K/AKT pathway might be involved in AURKA triggered angiogenesis procedure. In addition, AURKA overexpression impeded oxidative stress and subsequent following lipid peroxidation both in vitro and in vivo, indicating another protective mechanism of AURKA function in diabetic limb ischemia. The changes in lipid peroxidation biomarkers (lipid ROS, GPX4, SLC7A11, ALOX5, and ASLC4) in in vitro and in vivo were suggestive of the possible involvement of ferroptosis and interaction between AUKRA and ferroptosis in diabetic limb ischemia, which need further investigation.

**Conclusions:**

These results implicated a potent role of AURKA in diabetes-related impairment of ischemia-mediated angiogenesis and implied a potential therapeutic target for ischemic diseases of diabetes.

## Introduction

Critical limb ischemia (CLI), caused by arterial stenosis and occlusion, is the end stage of peripheral artery disease (PAD) (Gresele et al. 2011). In conjunction with the rise of the aging population and metabolic syndrome, the morbidity and mortality of PAD and CLI are increasingly climbing (Murabito et al. 2002). CLI is defined as resting pain or tissue necrosis with ulceration or gangrene in the setting of proven peripheral arterial occlusive disease (Hirsch et al. 2006). Among patients with CLI, up to 50% will undergo amputation and up to 20% will die from their disease (Fard et al. 2020). Among the numerous risk factors that predispose the development of CLI, diabetes mellitus is recognized as an independent risk factor, which is associated with both prevalence and severity. Hyperglycemia significantly contributes to the development of myopathy and microvascular disease, which determines muscle cell loss, endothelial dysfunction, and muscle fibrosis (Huysman and Mathieu 2009). The revascularization strategy is an effective y approach for CLI treatment (Cerqueira and Duarte 2020). Additionally, the outcome of revascularization in diabetic patients is not favorable, which increased the possibility of patients undergoing amputation (Al-Delaimy et al. 2004). Diminished angiogenic response to tissue damage and hypoxia in diabetes leads to a strong tendency to develop persistent foot ulcers (Brem and Tomic-Canic 2007). The lack of therapeutic options and the poor efficacy of diabetic CLI treatment drive the exploration of molecular mechanisms and angiogenesis pathways to better understand the detailed pathogenesis of CLI.

The kinome, encoded by approximately 2% of the human genome, is one of the largest homologous protein superfamilies consisting of 538 kinases (Manning et al. 2002). Mutation and dysregulation of protein kinases lead to multiple diseases and affect physiological activities (Greenman et al. 2007). The function of protein kinases in angiogenesis has been implicated in several diseases (Griner and Kazanietz 2007). Aurora Kinase A (AURKA) is a member of serein/threonine kinases that are expressed in multiple cell types (Yan et al. 2016). AURKA localizes to the centrosome and is required for centrosome maturation and division and mitotic spindle formation, thereby committing the cell to mitosis (Nigg 2001). Amplification of AURKA has been validated in various cancer types, including ovarian, gastric, and bladder cancer, and proposed to be an oncogene (Dutertre et al. 2002; Yan et al. 2016). Among the published reports on the pro-oncogenic effect of AURKA, the promotion of tumor angiogenesis is an important aspect. Downregulation of AURKA decreased cell proliferation and angiogenesis in neuroblastoma (Romain et al. 2014). Besides, AURKA has been reported to have a positive effect on vascular endothelial growth factor A (VEGFA) transcription (Dar et al. 2009). VEGFA is the potent pro-angiogenic factor for angiogenesis and is required for endothelial cell functions, such as proliferation, migration, and permeability (Burri et al. 2004). In human umbilical vein endothelial cells, the activation of AURKA is vital for the promotion of hypoxia-induced angiogenesis by modulating endothelial cell dynamics (Ki et al. 2020). In vascular disease, atherosclerosis, the potential role of AURKA on endothelial cell dynamics has been implied (Liu et al. 2019). In addition to its role in angiogenesis, in diabetes, AURKA inhibits high glucose-induced stem cell apoptosis and promotes repair of damaged skin repair in diabetic mice (Yin et al. 2020). These findings shed insights into the role of AURKA on angiogenesis in CLI associated with diabetes, which has not been reported in this field.

Oxidative stress is a common contributor to the progression of multiple diseases. Overproduction of oxygen free radicals leads to the progression of diabetes and vascular dysfunction (West 2000). In oxidative stress, AURKA has been identified as a major kinase to modulate cell proliferation (Wang et al. 2017). The knockdown of AUKRA could result in the accumulation of ROS, leading to cell apoptosis (Dawei et al. 2018). As a primary outcome of oxidative stress, the accumulation of lipid peroxidation products is prevalent in ischemic conditions (Ambrosio et al. 1991). Studies have illustrated that ferroptosis is the result of lipid peroxidation (Xie et al. 2016). The involvement of ferroptosis in endothelial cell dysfunction of vascular diseases has been implied (Bai et al. 2020), and ferroptosis could be modulated by overexpressed AURKA in non-small cell lung cancer (Li et al. 2022). Therefore, in our study, we aimed to explore the role of AURKA in the regulation of angiogenesis in diabetic CLI.

## Materials and methods

### Analysis of GSE datasets

Expression profiles of skeletal muscles from healthy controls and CLI patients were obtained from the Gene Expression Omnibus dataset GSE 120642. The screening criteria for differential gene expression analysis was |log2 fold change (log2FC)|> 2, *P* < 0.05. The list of human kinases used for the analysis was taken from Zhang et al. (2021) and the KinHub List of Human Kinases. The tree file of the human kinome was courtesy of Cell Signaling Technology and was presented using the web-based tool KinMap (Eid et al. 2017). The expression changes and log2FC were reflected by the colors and size of the squares. Gene expression correlation in muscle between VEGFA and differentially expressed kinase was performed using GEPIA datasets.

### Animal studies

All in vivo experiments were involved of 10 weeks male C57BL/6 mice (Liaoning Changsheng Biotechnology Co. Ltd., Liaoning, China), and approved by the First Affiliated Hospital of Zhengzhou University. Mice were housed in a 12-/12-h light/dark cycle with free access to standard mouse chow and water and acclimated to the facility 7 days before the experiment.

### Experimental models

#### In vivo Matrigel plug assay

Matrigel plug assay was conducted according to the previously published methods (Meng et al. 2018). Briefly, adenoviral vectors for AURKA overexpression (Ad-AURKA^OE^) and its negative control (Ad-AURKA^OE^) were constructed, propagated, and titered. Matrigel mixed with 50 ng/mL VEGF and 30 U/mL heparin was injected subcutaneously into mice together with Ad-AURKA^OE^ or Ad-NC^OE^. After a period of one week, the Matrigel plug was taken out for subsequent experimental manipulations. Hematoxylin and eosin staining was conducted to visualize the formation and infiltration of functional microvessels.

#### A diabetic mouse model with CLI

C57BL/6 mice were intraperitoneally administered with freshly prepared streptozotocin (STZ) solution (Shanghai yuanye Bio-Technology Co., Ltd., Shanghai, China, CAS. S17049) solution at a dose of 50 mg/kg body weight for five consecutive days to induce diabetes. Seven days after the first STZ administration, blood glucose levels were tested and only those mice with glucose levels above 15 mmol/L were included in the protocol. After the confirmation of diabetic mice, unilateral hind limb ischemia was surgically induced by femoral-saphenous artery-vein pair resection (Sarkar et al. 2009). For the gene transduction experiment, mice were anesthetized to induce hind limb ischemia using a procedure consisting of the ligation of both the proximal end of the femoral artery and the distal portion of the saphenous artery. Immediately, Ad-AURKA^OE^ or Ad-NC^OE^ (10^9^ plaque-forming unit) was delivered to the ischemic adductor muscle and gastrocnemius muscles (Shaikh et al. 2017). The blood flow of the ischemic and the contralateral feet was sequentially measured (at 0, 3, 7, 14, 21, and 28 days post-surgery) by laser Doppler Image, and the relative perfusion ratio between the ischemic foot and non-ischemic foot was calculated. The behavioral motor deficit was assessed using the Tarlov-motor scoring system (Westvik et al. 2009) at the above time points. At 21 days post-ischemia, gastrocnemius muscles from terminally anesthetized mice were frozen or paraformaldehyde fixed for further analysis.

#### Cell cultures and treatment

Human microvascular endothelial cells (HMEC-1) (iCell Bioscience Inc., Shanghai, China) were cultured in EGM-2MV medium (Lonza, Basel, Switzerland) supplemented with fetal bovine serum (FBS) and growth factors at 37 °C in the presence of 5% CO_2_ in the air. To mimic diabetes in vitro, HMEC-1 cells were subjected to endothelial basal medium (EBM-2, Lonza) containing 25 mmol/L d-glucose (Aladdin, Shanghai, China) (high glucose, HG). To simulate ischemia-induced tissue starvation, cells were cultured in medium supplemented with no additional growth factors (nutrient deprivation, ND) (Caporali et al. 2011). Cells incubated with d-Mannitol (normal glucose, NG) and EGM-2MV medium supplemented with FBS and growth factors served as controls.

Prior to cell seeding, cells were infected with prepared Ad-AURKA^OE^ or Ad-NC^OE^ at a multiplicity of infection of 10 for 48 h. Cells were then detached and re-seeded on plates for subsequent analyses under different stimuli as described above.

#### Cell permeabilization assay

Cell suspension (200 μL) with a density of 5 × 10^4^ cells/mL was added to the upper compartment of the Transwell chambers, and the lower compartment was filled with culture medium. After the establishment of confluent monolayers of cells, the medium was discarded and the washing procedures were repeated twice using PBS. A total of 100 μL fluorescein isothiocyanate (FITC)-labeled dextran (FITC-dextran, 1 mg/mL, Maokang Biotechnology, Shanghai, China) was added to the upper compartment, and 500 μL phosphate buffer saline (PBS) solution was added to the lower compartment. The cell permeability was assessed by measuring the fluorescence intensity of FITC-dextran from the upper compartment to the lower compartment.

#### Hematoxylin and eosin (HE) staining

HE staining was performed accordingly to a standard procedure on 5 μm thick Matrigel plug sections and gastrocnemius muscles. Slides were stained with hematoxylin (Solarbio, Beijing, China) and eosin (Sangon, Shanghai, China), according to standard procedure. After the staining was completed, the number of capillaries of gastrocnemius muscle sections was quantified. The recognition and quantification was referred by Zaccagnini et al. (2019).

#### Immunofluorescent (IF) staining

HMEC-1 cells were fixed with 4% paraformaldehyde and permeabilized with 0.1% Triton-X-100. The proliferation was visualized using bromodeoxyuridine (BrdU) staining. Slides were stained with anti-BrdU (1:100, ABclonal, Wuhan, China, #A20304), followed by Cy3-conjugated anti-rabbit antibody (1:200, Invitrogen, Carlsbad, CA, USA, #A27039). CD31 staining was performed on 5 μm thick Matrigel plug slides. Slides were stained with anti-CD31 (1:100, Santa Cruz Biotechnology, Santa Cruz, CA, USA, #Sc-376764), followed by Cy3-conjugated anti-mouse antibody (1:200, Invitrogen, #A21424). For functional vessel identification, mice were injected with FITC-labeled isolectin-B4 via tail vein, 30 min before sacrifice. Double IF staining was conducted on 5 μm thick gastrocnemius muscles. The perfused capillaries were labeled with anti-CD31 (1:100, Santa, #Sc-376764), followed by Cy3-conjugated anti-mouse antibody (1:200, Invitrogen, #A21424) and FITC-conjugated anti-rabbit antibody (1:200, Abcam, MA, USA, #AB6717). The density of small arteries (CD31^+^/α-SMA^+^) and capillaries (CD31^+^/α-SMA^−^) were double stained with anti-CD31 (1:100, Santa, #Sc-376764) and anti-α-SMA (1:100, ABclonal, A17910), followed by Cy3-conjugated anti-mouse antibody (1:200, Invitrogen, #A21424) and FITC-conjugated anti-rabbit antibody (1:200, Abcam, #AB6717).

#### Immunohistochemical (IHC) staining

IHC staining was performed accordingly to a standard procedure on 5 μm thick gastrocnemius muscles. Slides were stained with Desmin (1:100, ABclonal, #A3736) or 4-HNE (1:100, Abcam, #AB48506), followed by HRP conjugated anti-mouse antibody (1:500, ThermoFisher, Pittsburgh, PA, USA, #31430) or anti-rabbit antibody (1:500, ThermoFisher, #31460).

#### Cell proliferation and tube formation assay

Cell suspension with a density of 5 × 10^4^ cells/mL was seeded into 96-well plates and cultured respectively for 12, 24, and 48 h. After incubation with 3-(4,5-dimethylthiazol-2-yl)-2,5-diphenyltetrazolium bromide (MTT, KeyGEN, Nanjing, China) for 4 h at the indicated time points, the absorbance of each well at 490 nm was recorded to represent cell viability.

Cells (100 μL, 2 × 10^5^ cells/mL), after indicated treatments, were seeded on plates coated with Matrigel. Tube formation was observed at 8 h under light microscopy (Olympus, Tokyo, Japan). The tube formation ability was evaluated in terms of the length and bifurcation points of the tube structure.

#### Scratch-wound assays

Cells grown in six-well plates were treated as per experimental requirements and cultured with 1 μg/mL Mitomycin C (Sigma-Aldrich, St. Louis, MO, USA) for 1 h prior to the scratch procedure. A liner scratch was created in the confluent cell monolayer using a sterile 200 μL pipette tip. Representative images were obtained at 24 h under a microscopy, and the migration capacity was defined as the blank distance at 0 h and at 24 h.

#### ROS and lipid ROS detection assay

Cultured cells were digested with 0.25% Trypsin–EDTA and centrifuged at 140*g* for 5 min, and then the cell precipitate was washed and re-harvested. 2′,7′-dichlorofluorescein diacetate (DCFH-DA, KeyGEN), diluted as 1:1000 in serum-free medium. Cells were pre-incubated with appropriate volume DCFH-DA solution for 30 min in the dark. DCFH-DA that failed to enter cells was removed with PBS. Cellular ROS was fluorometrically monitored by flow cytometry. The detection of lipid ROS was carried out in the same manner described above, except that the fluorescent probe C11-BODIPY (581/591) (Gibco, Grand Island, NY, USA) was used. ROS level in tissues were observed using a ROS staining kit (BestBio, Shanghai, China) following the manufacturer's protocol.

### Immunoblotting

Tissues or cells were solubilized in RIPA lysis buffer (Beyotime, Shanghai, China) containing protease inhibitors (Beyotime), and the obtained lysates were prepared by centrifugation at 10,000*g* for 3 min at 4 °C. The protein concentration of the lysates was quantified using a Bicinchoninic acid assay kit (Beyotime). Equal amounts of denatured- protein samples were separated on SDS–polyacrylamide gel electrophoresis (PAGE) (Beyotime) and transferred on polyvinylidene difluoride (PVDF) membranes (ThermoFisher). The membranes were incubated with blocking buffer (5% [m/v] bovine serum albumin (BSA) in Tris-buffered Saline-Tween 20), following primary and secondary horseradish peroxidase-conjugated antibodies incubation. All antibodies were diluted in blocking buffer: anti-AURKA (1:500, ABclonal, #A2121), anti-VEGFA (1:1000, ABclonal, #A5708), anti-GPX4 (1:1000, ABclonal, #A11243), anti-SLC7A11 (1:1000, ABclonal, #A13685), anti-ACSL4 (1:1000, ABclonal, A16848), anti-ALOX5 (1:1000, ABclonal, A2877), anti-Cyclin B1 (1:500, ABclonal, A2056), anti-CDK1 (1:500, ABclonal, A0220), anti-p-PI3K (1:1000, ABclonal, AP0427), anti-PI3K (1:1000, ABclonal, A4992), anti-p-AKT (1:1000, Affinity, AF0016), anti-AKT (1:1000, Affinity, AF0836), anti-VEGFR2 (1:1000, Affinity, AF6281), anti-β-actin (1:2000, Proteintech, Wuhan, China, #60008-1-Ig), goat anti-mouse IgG-HRP (1:10000, Proteintech, #SA00001-1), goat anti-rabbit IgG-HRP (1:10000, Proteintech, #SA00001-2).

### RNA isolation and real-time quantitative PCR (RT-qPCR) analysis

Total RNA was isolated using TRIpure reagent (BioTeke, Beijing, China) in strict accordance with the manufacturer’s instructions. All extracted RNA was subjected to Nanodrop 2000 (ThermoFisher) to verify sample concentration and purity before cDNA synthesis. The cDNA was synthesized using M-MLV reverse transcriptase (Beyotime) in a total 20 μL reaction buffer containing random primers, oligo (dT)15 primers at 25 °C for 10 min, 42 °C for 50 min, and 80 °C for 10 min. The prepared cDNA samples were tested to examine the expression of genes using Exicycler™ 96 Real-Time Quantitative system (BIONEER, Daejeon, Korea) in the presence of SYBR Green Master Mix (Solarbio). Primers sequences from 5′ to 3′ (synthesized by GenScript, Nanjing, China) were listed as follow: homo-AURKA (ACCTTCGGCATCCTAATA and AGCATGTACTGACCACCC); homo-VEGFA (TCACCAAGGCCAGCACATAG and GGGCACCAACGTACACGCT); homo-GPX4 (GAAGCAGGAGCCAGGGAGT and ACGCAGCCGTTCTTGTCG); homo-SLC7A11 (CCCTTTCCCTCTATTCGG and ACCTGGGTTTCTTGTCCC); homo-ACSL4 (GCATTCCTCCAAGTAGACC and ATGAGCCAAAGGCAAGT); homo-ALOX5 (GACAAGCCCTTCTACAACGA and CCATCCCTCAGGACAACC); mus-AURKA (CTTTCCCTGACTTTGTGAC and ATTTGCTGGTTGGCTCTT); mus-GPX4 (CAACCAGTTTGGGAGGC and CTTGGGCTGGACTTTCAT); mus-SLC7A11 (TTGGAGCCCTGTCCTATGC and CGAGCAGTTCCACCCAGAC).

### Detection assay kit

The content of malondialdehyde (MDA) (in cells or gastrocnemius muscles), glutathione (GSH) (in cells or gastrocnemius muscles), hemoglobin (in matrigel plug), and VEGFA (in gastrocnemius muscles) was detected using corresponding commercial detection kit or ELISA kit, following the manufacturer’s recommendation. Cell cycle detection was conducted using commercial cell cycle detection kit (KeyGEN) according to protocol’s instruction by flow cytometer.

### Statistics analysis

Statistical analyses were conducted using Graphpad Prism 8.0. All results are shown as scatter and mean ± standard deviation (SD), except behavioral deficits scores (each dot represented the corresponding scores per animal). Each experiment contained at least three biological replicates, of each performed in triplicate. One- and two-way analysis of variance (ANOVA) with Tukey’s post-hoc tests were utilized to analyze statistical differences between multiple groups. Student’s t-test was utilized to analyze statistical differences between two groups. *P* value < 0.05 was recognized as significance (^*^P < 0.05, ^**^P < 0.01).

## Results

### Expression of kinases in CLI

**Fig. 1:**
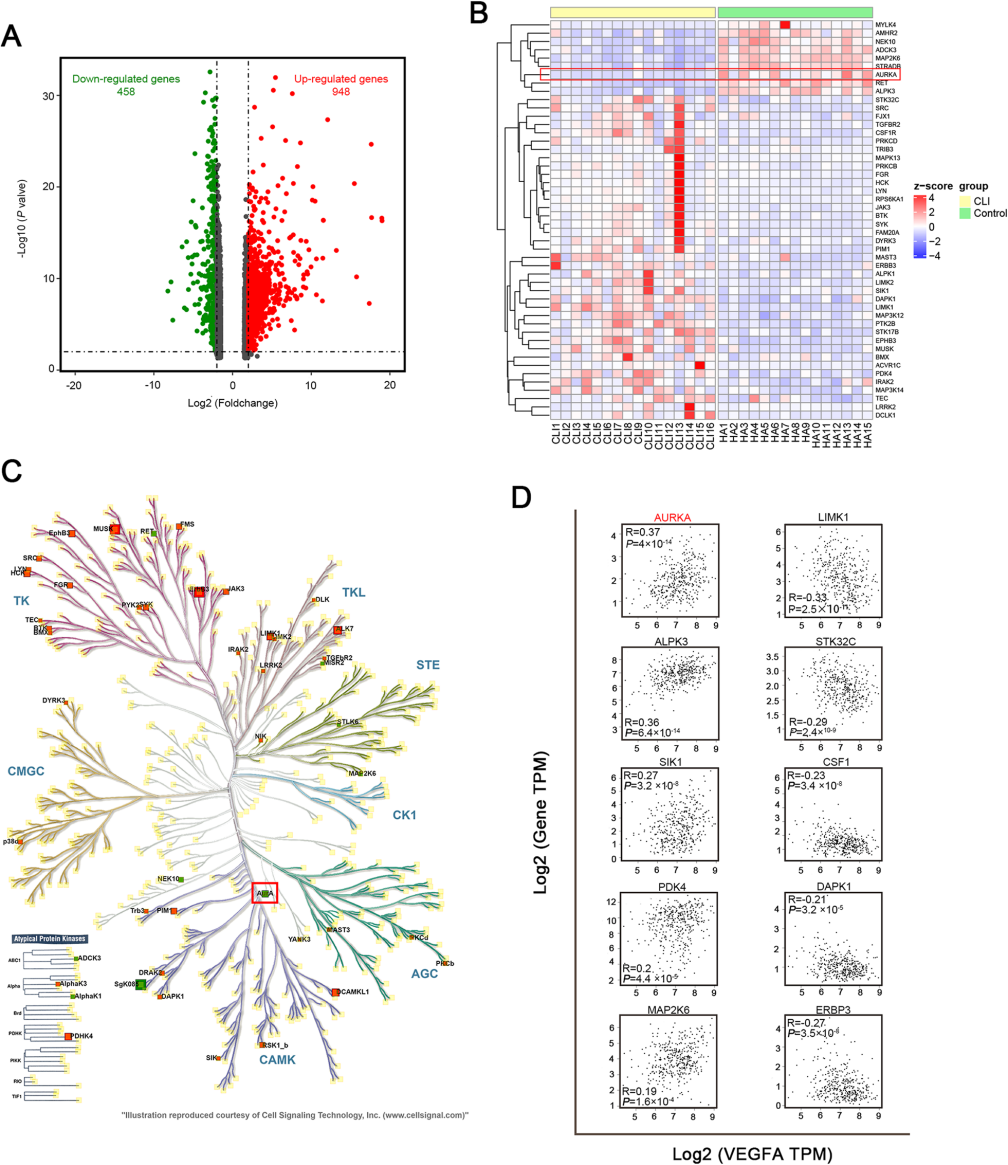
Expression of kinases in CLI. The differential genes in CLI were analyzed using GSE120642 dataset, and shown in volcano (**A**). The changes of differential expressed kinases were exhibited in heat map (**B**). Distribution of changed kinases levels was shown over the human kinome tree (**C**). Increased levels of kinases were in red, and decreased were in green. The square sizes reflected Log2 Fold change (Log2FC) (**C**). Verification of the correlation of VEGFA and the differentially expressed kinase genes using GEPIA (**D**)

To gain insight into the differential gene expression in CLI patients, the RNA-seq dataset was obtained from GSE120642. A population of 1406 differentially expressed genes was screened (Fig. 1A), from which 48 kinases (among total 538 human kinases reported by Zhang et al. (2021) were selected for further study (Fig. 1B). The heat map showed the expression of the selected kinases in the dataset GSE120642 with 15 healthy controls and 16 CLI patients. Nine of these kinases were significantly downregulated and 39 were upregulated (Fig. 1B, C). To further investigate the possible association of kinase genes and VEGFA (the most important angiogenesis factor), the correlation between the parameters was analyzed using GEPIA. As show in Fig. 1D (shown the top 10 positive/negative correlations), the AURKA had the relatively strong correlation among these genes. Upon reviewing the literature, we noticed that AURKA was closely associated with tumor angiogenesis with positive effect on VEGFA transcription and glucose metabolism. We, therefore, selected AURKA gene to study in diabetic limb ischemia.

### Overexpression of AURKA enhances the proliferation of HMEC-1 after high glucose and nutrient deprivation injury

**Fig. 2:**
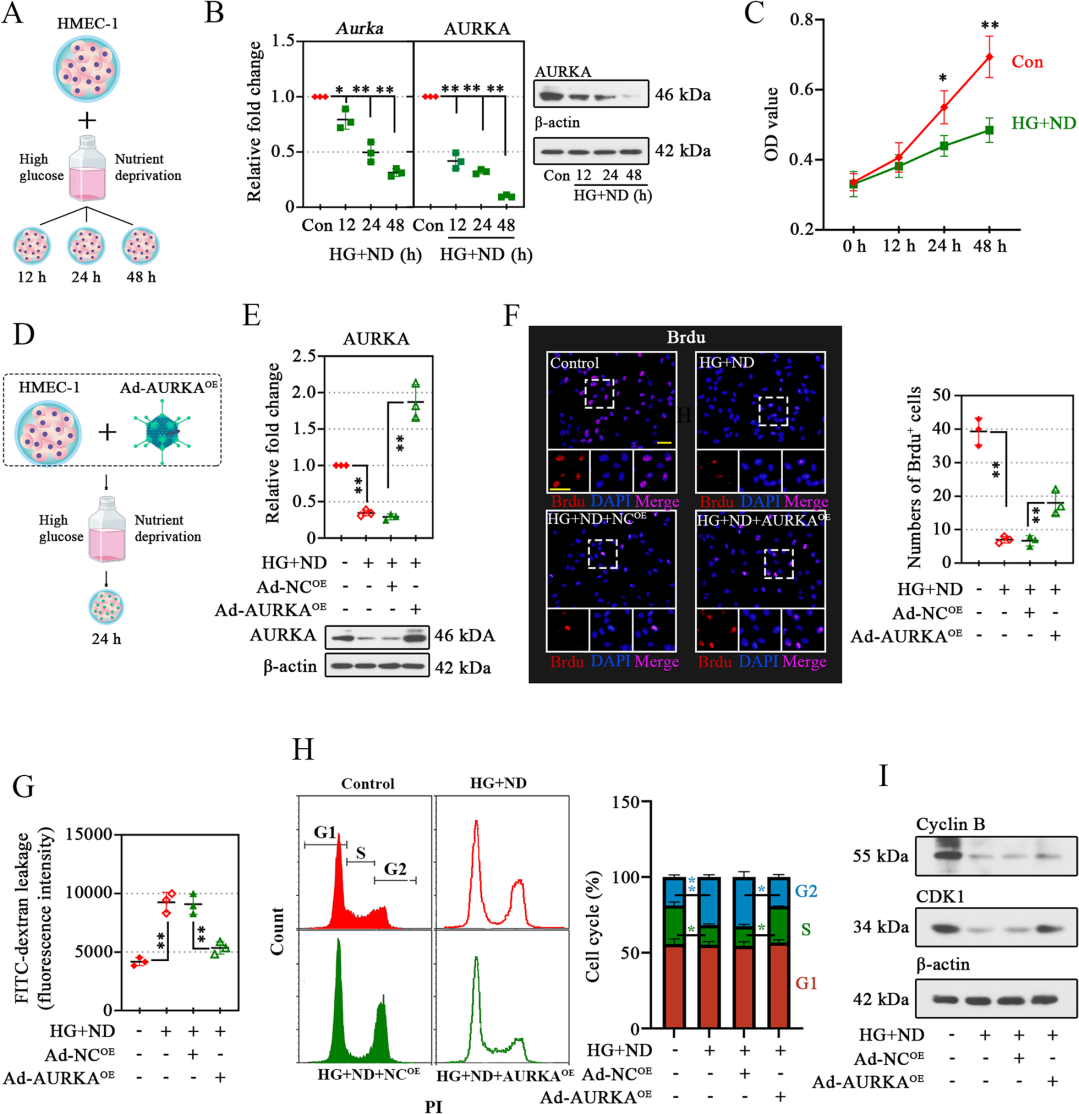
Overexpression of AURKA enhances the proliferation of HMEC-1 after high glucose and nutrient deprivation injury. Human microvascular endothelial cell (HMEC-1) cells were incubated with high glucose condition (HG) with nutrition deprivation (ND) for 12, 24, or 48 h (**A**). After that, the expression validation of Aurora kinase A (AURKA) (**B**) and cell viability were analyzed (**C**). AURKA was overexpressed via transduction with adenoviral vectors and treated with medium for 24 h (**D**), and the transduction efficiency was confirmed by the protein expression of AURKA (**E**). After 24 h of transfection, the cell proliferation was evaluated using bromodeoxyuridine (Brdu) labeling, and its quantification data were shown on the right. Pink spots were scored as positive-stained Brdu (scale bar, 50 μm) (**F**). The endothelial permeability of cells was tested using fluorescein isothiocyanate (FITC)-dextran (**G**). Flow cytometry analysis on cell cycle was analyzed (**H**), and the protein expression of cell cycle related proteins Cyclin B1 and CDK1 was measured using Western blotting (**I**). Data were represented as mean ± standard deviation (SD). ^*^P < 0.05, ^**^P < 0.01. Each dot indicated a biological replicate, n = 3

The positive effects of AURKA on endothelial cell function and angiogenesis have been established in cancer (Yan et al. 2016). Microvascular endothelial cells are integral players in the processes of angiogenesis. Here, we sought to confirm the change of AURKA expression under a mimicked diabetes and ischemia-induced tissue starvation environment in vivo. HMEC-1 cells were cultured in d-Mannitol or 25 mmol/L d-glucose (HG) with low growth factors (ND) for different time points. d-Mannitol supplied equal osmotic pressure without metabolization in cells (Fig. 2A). Exposure of HMEC-1 cells to HG and ND conditions decreased AURKA expression (Fig. 2B) and impaired its proliferative ability (Fig. 2C), and these injuries were in a time-dependent manner. These data suggested that microvascular endothelial cells expressed AURKA and that expression was consistently impaired under HG and ND conditions. The AURKA was overexpressed in HMEC-1 cells to validate its functional consequence (Fig. 2D, E). Increased proliferation and invasive capacity of endothelial cells are key processes in angiogenesis that are also associated with the maintenance of microvascular permeability barriers (Battle et al. 2006). Under HG and ND conditions, the proliferation (Fig. 2F) capacities of HMEC-1 cells were impaired, yet these defects were corrected with the addition of Ad-AURKA^OE^ (Fig. 2F). Besides, the permeability of HMEC-1 cells was rescued by AURKA overexpression (Fig. 2G). Given the effect of AURKA on cell cycle, we assessed the ability the AURKA on cell cycle in HMEC-1 cells. As the results shown, the cell number was increased in cells under HG and ND condition at G2/M phase (Fig. 2H); while, AURKA overexpression promoted the cell cycle arrest in G2/M phase (Fig. 2H). Besides, the overexpression of AURKA increased the protein expression of Cyclin B and CDK1 (Fig. 2I). All these indicated that AURKA significantly promoted cell proliferation of HMEC-1 cells.

### Overexpression of AURKA exerts a promoting effect on migration and tube formation ability of microvascular endothelial cells

**Fig. 3:**
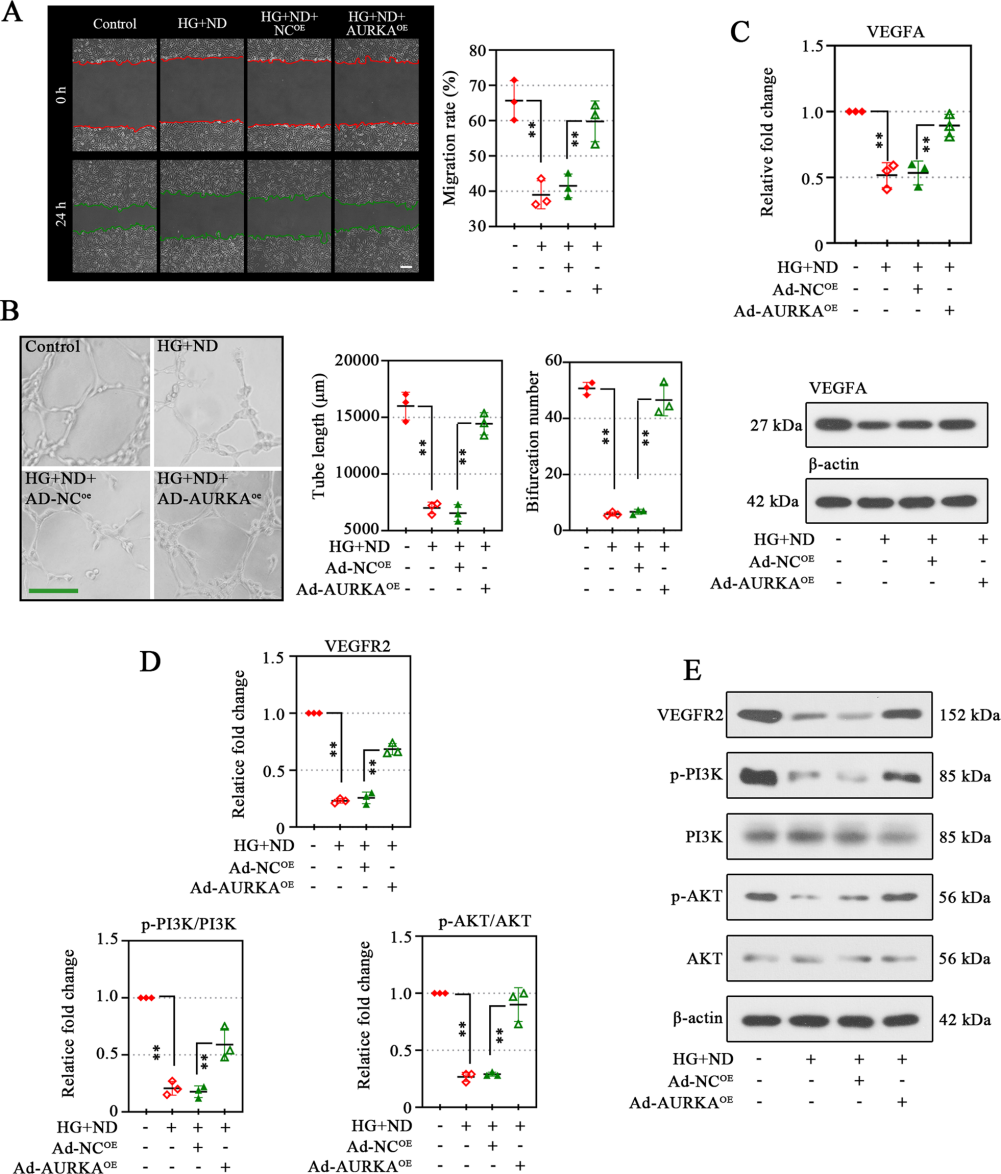
Overexpression of AURKA exerts a promoting effect on migration and tube formation ability of microvascular endothelial cells. The migration of cells was analyzed by scratch assay (scale bar, 200 μm) (**A**), and the percentage of the scratch migration rate was shown on the right panel (**A**). Red and green line indicated the boundary lines of scratch (**A**). Tube formation assay (**B**) was conducted to measure the tube formation ability of HMEC-1 cells with AURAK overexpression, and the tube-like structure length and bifurcation point numbers were quantified as shown on the right. Western blot assay were used to detect the expression of vascular endothelial growth factor A (VEGFA) in HMEC-1 cells (**C**). Protein levels of Vascular Endothelial Growth Factor Receptor 2 (VEGFR2), phosphatidylinositol 4,5-Bisphosphate 3-Kinase (PI3K), phosphorylated (p)-PI3K, serine-threonine protein kinase (AKT), and p-AKT were analyzed using Western blotting (**D**, **E**). Data were represented as mean ± SD. ^**^P < 0.01. Each dot indicated a biological replicate, n = 3

Endothelial migration is an initial step in angiogenesis. Overexpression of AURKA promoted the migration-restraining effect of HG and ND (Fig. 3A). The angiogenic ability of AURKA on HMEC-1 cells was confirmed by tube formation assay, which could directly show the vascular-like structures. In AURKA-overexpressed HMEC-1 cells, the elevated tube formation capacity was observed compared with its negative controls (Fig. 3B, left), accompanied by increased node number and total tube length (Fig. 3B, right). Thus, AURKA might be involved in the promotion of microvascular endothelial cell migration and tube formation, thus promoting angiogenesis. Angiogenesis is initiated by the activation of resting endothelial cells in response to angiogenic stimuli. VEGFA is the potent pro-angiogenic factor essential for the development of angiogenesis. An obvious decrease in VEGFA levels was observed in HMEC-1 cells during HG and ND stimulation (Fig. 3C), and these expression changes in HMEC-1 cells with Ad-AURKA^OE^ treatment were significantly up-regulated (Fig. 3C). In the signaling network relevant to angiogenesis, we measured the VEGFR2/PI3K/AKT. Overexpression of AURKA significantly increased the expression of VEGFR2, p-PI3K, and p-AKT under HG and ND conditions (Fig. 3D, E).

### AURKA overexpression protects cells against oxidative stress upon high glucose and nutrient deprivation stimulation

**Fig. 4:**
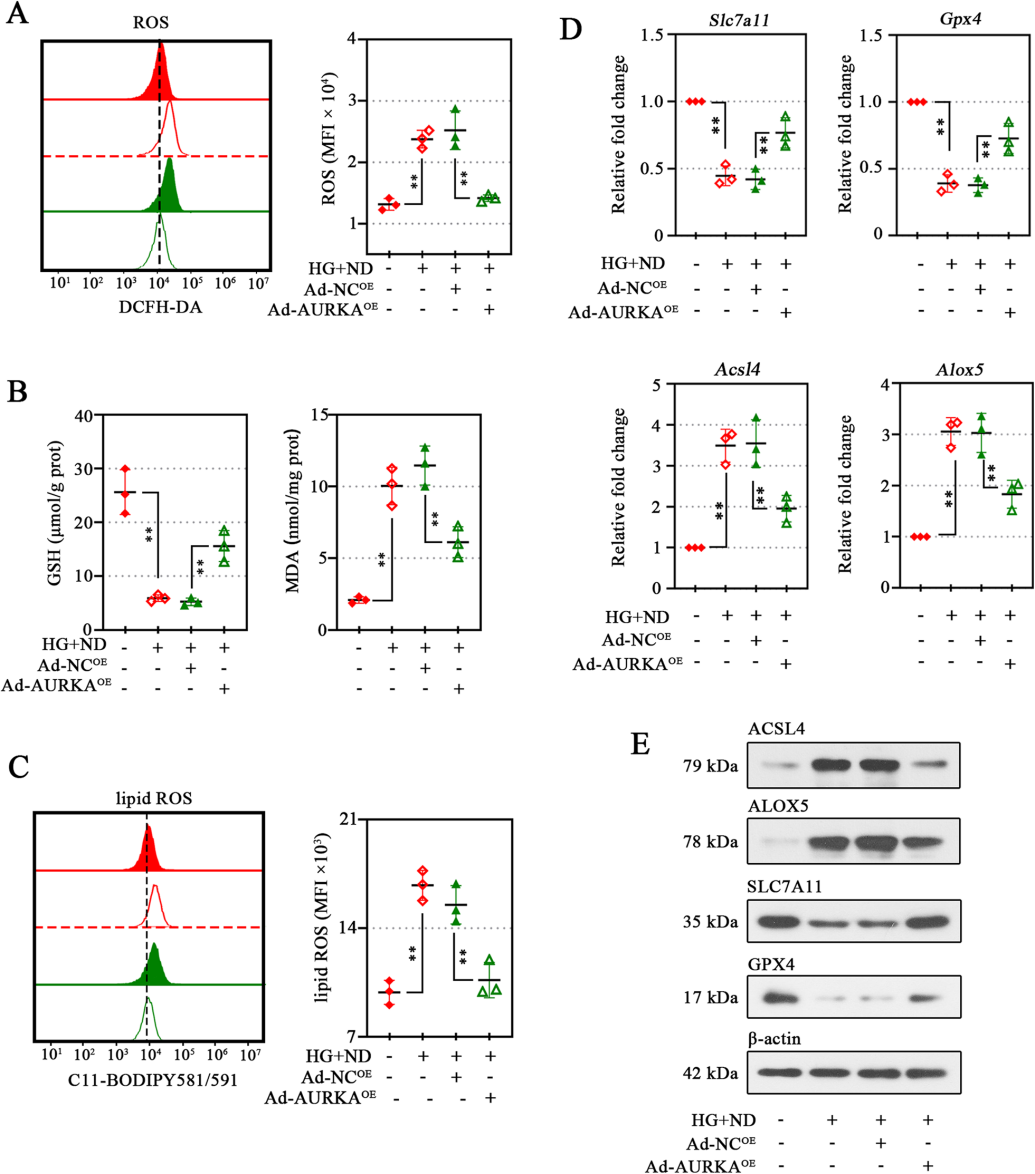
AURKA overexpression protects cells against oxidative stress upon high glucose and nutrient deprivation stimulation. Detection of ROS production in HMEC-1 cells using the ROS-specific fluorescent probe 2,7-dichlorodihydrofluorescein diacetate (DCFH-DA) (**A**, left), and quantification of fluorescence intensities in indicated cells was displayed on the right (**A**). MFI, Mean fluorescence intensity. Relative levels of glutathione (GSH) (**B**) and malondialdehyde (MDA) (**B**) were examined in the cells. Detection of ROS production in HMEC-1 cells using the lipid ROS-specific fluorescent probe C11-BODIPY 581/591 and quantification of fluorescence intensities in indicated cells was displayed on the right (**C**). RT-qPCR (**D**) and Western blot (**E**) showed the levels of Solute Carrier Family 7 Member 11 (SLC7A11), Glutathione Peroxidase 4 (GPX4), Acyl-CoA Synthetase Long Chain Family Member 4 (ACSL4), and Arachidonate 5-Lipoxygenase (ALOX5) in indicated cells. Data were represented as mean ± SD. ^**^P < 0.01. Each dot indicated a biological replicate, n = 3

Ischemia, including the high glucose, triggers the generation of reactive oxygen species (ROS) in vascular endothelial cells, leading to impaired cell structure and function (Battle et al. 2006). We found that AURKA overexpression rescued the increment of ROS (Fig. 4A) and MDA (Fig. 4B), as well as the GSH activity (Fig. 4B) induced by HG and ND. Lipid peroxidation served as the frequent injury resulting from the action of ROS (Bielski et al. 1983), leading to cell injury and death. We, therefore, investigated whether AURKA might be involved in lipid peroxidation. To this end, we first analyzed the endogenous lipid peroxidation levels. Not surprisingly, the HG and ND conditions induced provoke lipid peroxidation in cells (Fig. 4C). Whereas upon AURKA overexpression, the lipid-ROS levels were significantly decreased (Fig. 4C). Further, the drastic increase of Solute Carrier Family 7 Member 11 (SLC7A11) and Glutathione Peroxidase 4 (GPX4) levels (potent negative regulators of lipid peroxidation) and decrease in Arachidonate 5-Lipoxygenase (ALOX5) and Acyl-CoA Synthetase Long Chain Family Member 4 (ACSL4) (potent positive regulators of lipid peroxidation) induced by AURKA overexpression (Fig. 4D, E). These results suggested the potential capacity of AURKA on lipid peroxidation in these cells.

### AURKA overexpression enhances neovascularization in vivo

**Fig. 5:**
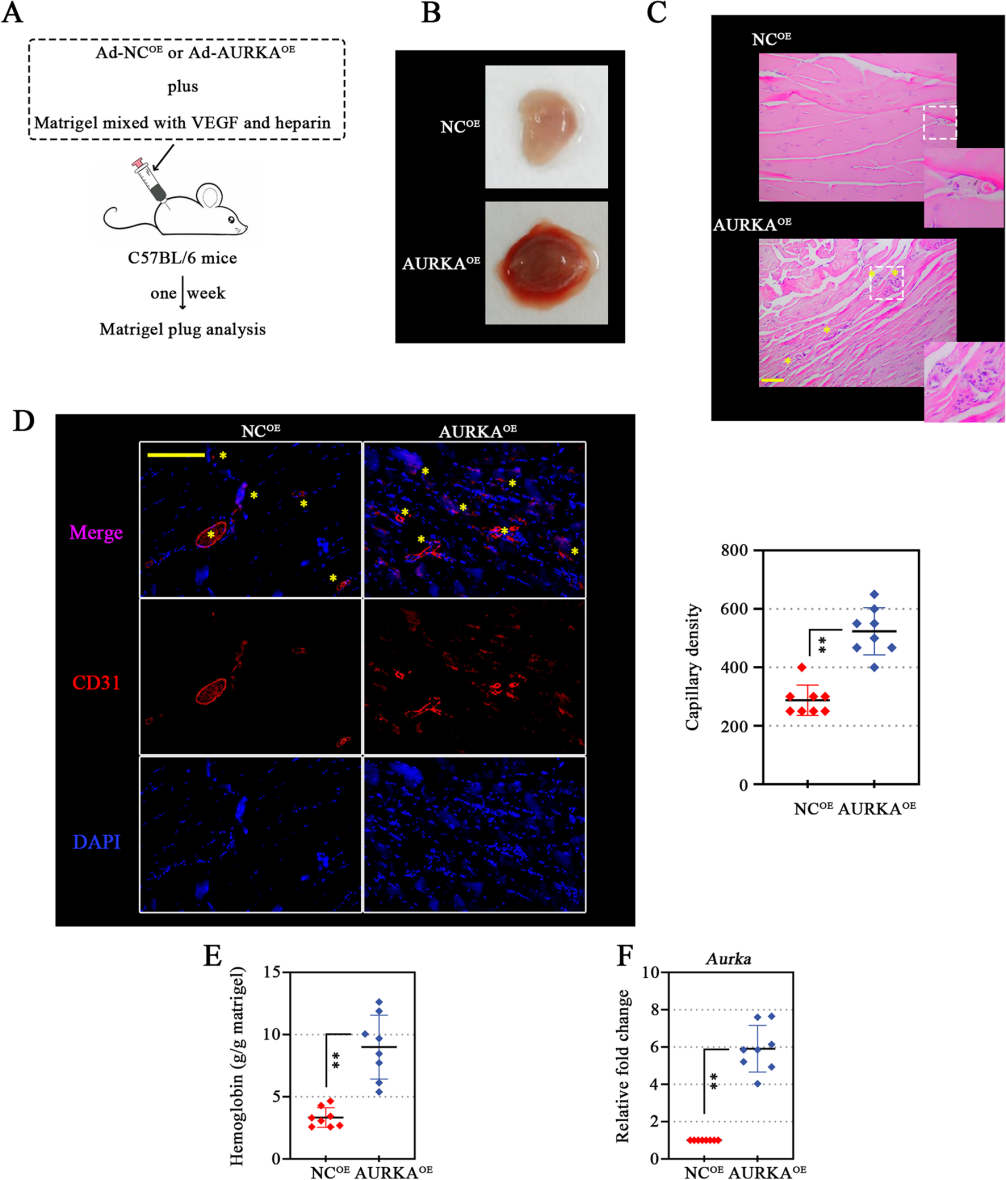
AURKA overexpression enhances neovascularization in vivo. In vivo matrigel plug implant model using C57BL/6 mice was performed by subcutaneous injection of Matrigel mixed with AURKA overexpressing-adenovirus (AURKA^OE^) or its negative control (NC^OE^) (**A**). One week after, a gross matrigel plug image was taken (**B**). Slides of each matrigel plug were stained with hematoxylin and eosin (H&E) to observe angiogenesis response (asterisk marks) in vivo (**C**) (scale bar, 100 μm). Slides of each matrigel plug were subjected to CD31 immunofluorescence staining to visualize (left) and quantify (right) capillary density (**D**). Pink spots (asterisk marks) were scored as positive-stained CD31 (scale bar, 200 μm) (**D**). The hemoglobin concentration of matrigel plug was investigated, reflecting new blood vessels formation (**E**). The mRNA expression of AURKA was further validated in matrigel plug (**F**). Data were represented as mean ± SD. ^**^P < 0.01. Each dot indicated a biological replicate, n = 8

To determine whether the effects of AURKA on microvascular endothelial cells in vitro translate into angiogenesis promotion in vivo, we investigated the effects of AURKA in the murine Matrigel plug model (Fig. 5A). Overexpressed AURKA was validated in the plug (Fig. 5F). The Matrigel plug harvested from negative control mice was relatively pale with mostly sparse blood aggregates (Fig. 5B, top). On the other hand, the Matrigel plug retrieved from AURKA-overexpressing mice was dark red and opaque (Fig. 5B, bottom), indicating the presence of significant blood aggregates. The elevated biochemical indices of hemoglobin (a marker of vascularization) proved the higher vessels vascularity with AURKA overexpression (Fig. 5E). The new vessel formation in plugs enhanced by AURKA overexpression was also confirmed by H&E staining (Fig. 5C). The intensity of the vessel was visualized and quantified by IF staining, using anti-CD31 (endothelial cell marker). The plug containing the Ad-AURKA^OE^ remarkably increased the vessel density compared to the negative control (Fig. 5D). These data supported the positive regulatory effect of AURKA on angiogenesis in vivo.

### AURKA overexpression promotes blood flow recovery in a mouse model of diabetic hind limb ischemia

**Fig. 6:**
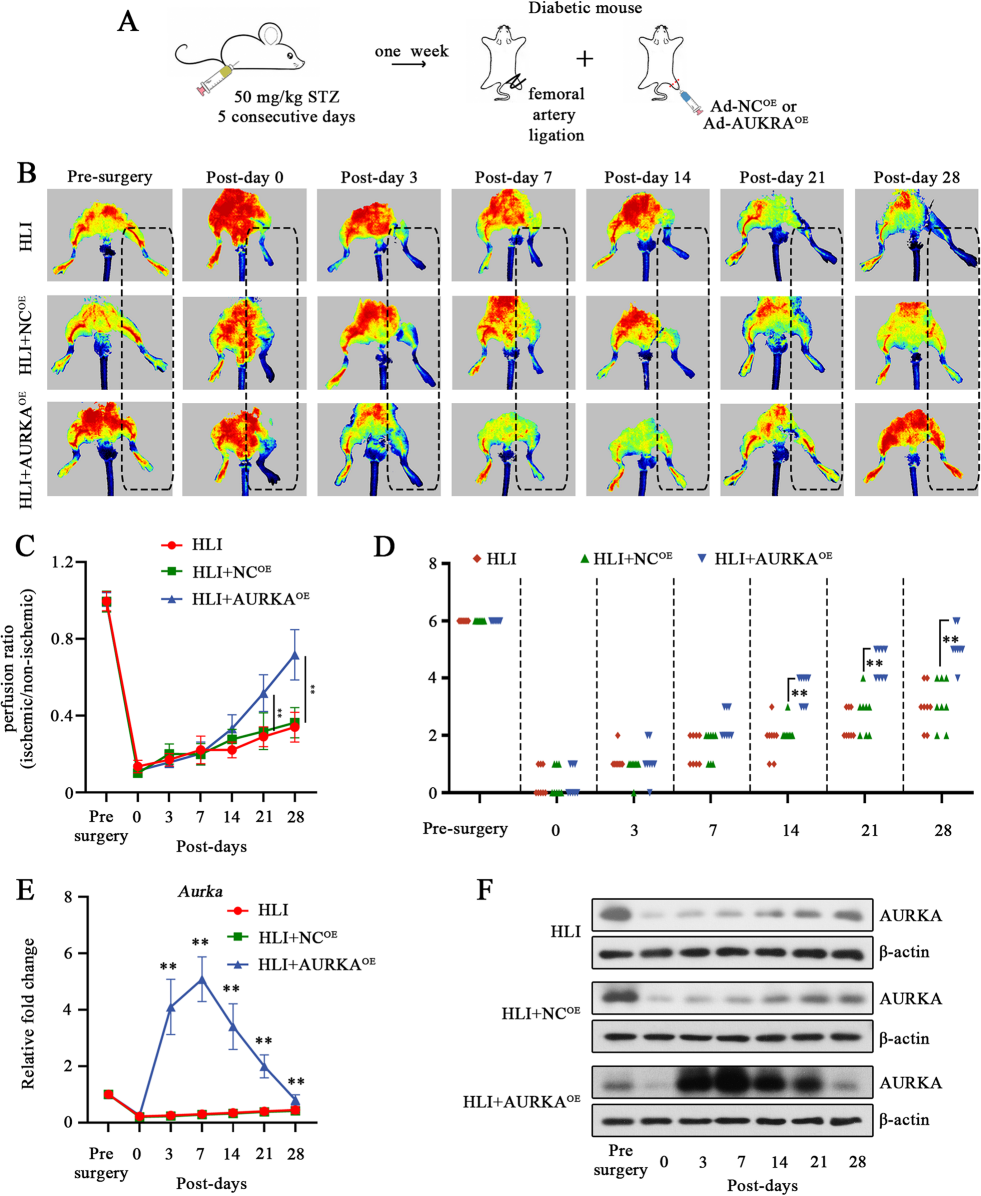
AURKA overexpression promotes blood flow recovery in a mouse model of diabetic hind limb ischemia. To determine the role of AURKA in post-ischemic angiogenesis in vivo*,* ischemia injury and blood recovery were assessed in diabetic mice with critical limb ischemia (established by STZ injection and femoral artery ligation) (**A**). Blood perfusion (**B**) and the quantitative analysis (**C**) of hind-limb was measured at indicated time point using laser Doppler perfusion imaging (LDPI) after intramuscular injection of AURKA overexpressing-adenovirus (AURKA^OE^) or its negative control adenovirus(NC^OE^). Behavior function was assessed according to Tarlov score at indicated time-point. The time dependent expression changes of AURKA in gastrocnemius muscle was examined by RT-qPCR (**E**) and Western blotting (**F**). For **C** and **E**, data were represented as mean ± SD. For **D**, scatter plot represented the Tarlov score for individual mouse. ^**^P < 0.01. Each dot indicated a biological replicate, n = 8

Next, we explored whether AURKA could exert its beneficial effect in the HLI model with diabetes (Fig. 6A). Diabetic mice overexpressing AURKA had enhanced blood flow recovery in response to left femoral artery ligation surgery (Fig. 6B). On days 21 and 28, the perfusion ratio in the ischemic limb had recovered to nearly 50% and 70%, respectively, and that of the negative control group has converted to nearly 30% (Fig. 6C). Meanwhile, the behavioral deficits were rescued in AURKA overexpressed mice based on Tarlov-Scoring system (Fig. 6D). According, the overexpression AURKA expression induced by adenovirus in the gastrocnemius muscle was confirmed. As Fig. 6E, F shown, the AURKA was significantly increased after injection at day 3, reached a peak on day 7, and then gradually declined at a subsequent time points (Fig. 6E, F). Based on these observations, we might conclude that AURKA plays a potent role in blood flow recovery under diabetes and ischemia.

### AURKA overexpression protects muscle against diabetic ischemia injury

**Fig. 7:**
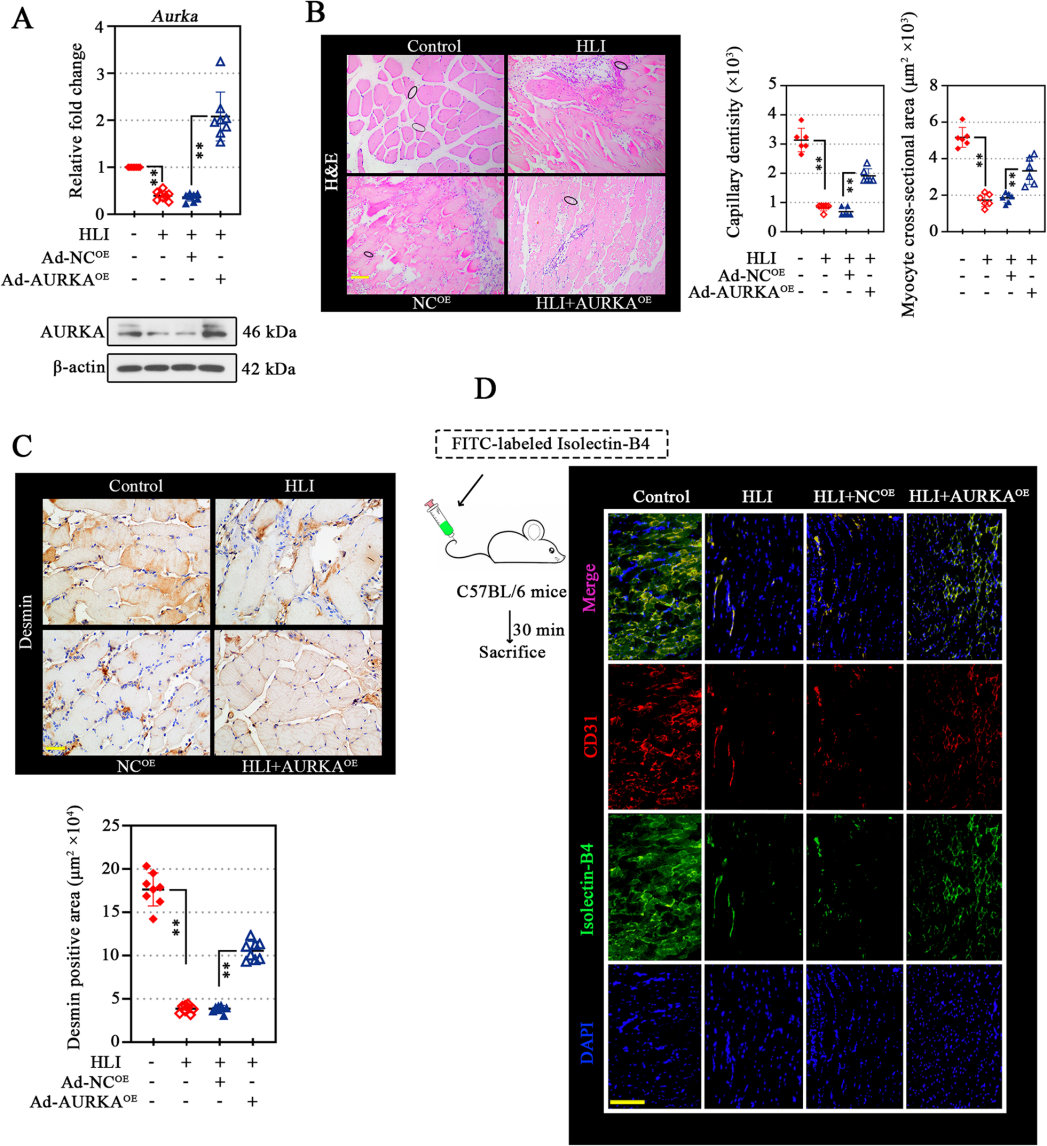
AURKA overexpression protects muscle against diabetic ischemia injury. After 21 days of modeling, the AURKA mRNA (bar graph) and protein (bands) levels in gastrocnemius muscle tissues from all experimental groups were validated (**A**). Pathologic changes in gastrocnemius muscle tissues were evaluated using H&E staining (**B**, left), and the capillary density and myofiber cross-sectional area were determined (**B**, right). The black circle indicated capillaries. Scale bar, 100 μm (**B**). Immunohistochemistry (IHC) staining of Desmin on gastrocnemius muscle tissues to visualize muscle regeneration (**C**, upper), and the Desmin^+^ area was measures (**C**, lower). Scale bar, 50 μm. FITC-labeled Isolectin-B4 (50 μL) was injected via tail vein thirty minute before the mice were sacrificed. Identification of perfused blood vessels and capillaries in the gastrocnemius muscle was observed by dual immunofluorescence with Isolectin-B4 and CD31. Scale bar, 200 μm (**D**). Data were represented as mean ± SD. ^**^P < 0.01. Each dot indicated a biological replicate, n = 8

The functional and morphological recovery of injured muscle is an important manifestation of angiogenesis (Deveci et al. 2002). In the gastrocnemius muscle, the AURKA expression was confirmed (Fig. 7A). The representative H&E stained sections of gastrocnemius muscle shown obvious histologic changes in each group (Fig. 7B). The capillary density was measured to determine the angiogenesis in gastrocnemius muscle. In AURKA-overexpressing gastrocnemius muscle under diabetic ischemia, the capillary density was significantly increased relative to the respective negative controls (Fig. 7B). The measurement of muscle fiber cross-sectional area in frozen sections of muscle is one of the most accurate approaches for the ex-vivo assessment of muscle atrophy. The gastrocnemius muscle of HIL-operated mice exhibited an increased area of atrophic fibers (Fig. 7B), as evidenced by a reduction in the cross-sectional area of muscle fibers (Fig. 7B). In contrast, an increase in the cross-sectional area of ischemic gastrocnemius muscle fibers was observed in diabetic mice subjected to AURKA overexpression (Fig. 7B). Effective muscle regeneration contributes to muscular atrophy restoration (Schiaffino et al. 2013). Desmin, an intermediate filament protein, is almost expressed during muscle regeneration processes (Gallanti et al. 1992). By detecting the Desmin expression by IHC (Fig. 7C), the enlarged Desmin-positive regions (positive staining of Desmin regardless of staining intensity) in the gastrocnemius muscle demonstrated the abundant regenerating of muscles in mice with AURKA overexpression under diabetic ischemia. Thus, the overexpression of AURKA appeared to enhance gastrocnemius muscle regeneration and improved injured muscle recovery. To better understand how AURKA overexpression restored distal perfusion in mice, the reperfusion of the vascular was examined, and FITC-labeled Isolectin-B4 was perfused systemically via tail vein injection 30 min before sacrifice (Fig. 7D). CD31 positive staining indicated vessels, and CD31-positive vessels with the co-localization lectin-B4 were recognized as well perfused blood vessels. As shown in Fig. 7D, the functional perfused vessels were markedly increased upon AURKA overexpression in the ischemic gastrocnemius muscle of diabetic mice.

### AURKA overexpression improved angiogenesis in ischemic gastrocnemius muscle of diabetic mice

**Fig. 8:**
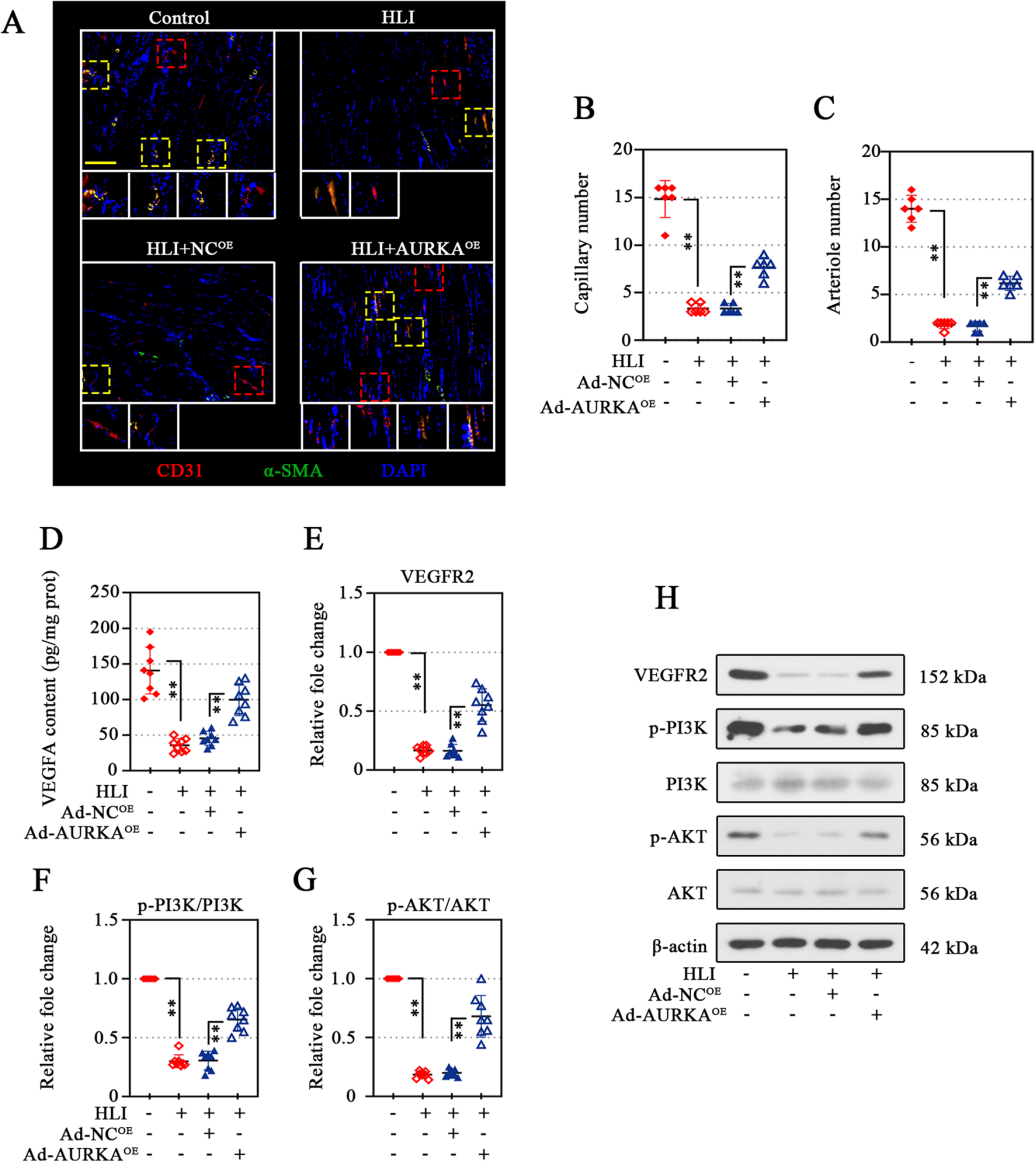
AURKA overexpression improved angiogenesis in ischemic gastrocnemius muscle of diabetic mice. After 21 days of modeling, dual-immunofluorescence analysis in gastrocnemius muscle tissues was conducted to visualize CD31 and α-smooth muscle actin (α-SMA). Scale bar, 200 μm (**A)**. The capillaries (CD31^+^/α-SMA^−^, red box identification) (**B**) and arterioles (CD31^+^/α-SMA^+^, yellow box identifications) (**C**) was quantified. The VEGFA content of gastrocnemius muscle tissues was determined (**D**). Protein levels in gastrocnemius muscle tissues of VEGFR2 (**E**), p-PI3K (**F**), PI3K (**F**), p-AKT (**G**), and AKT (**G**) were analyzed using Western blotting (**E**–**H**). Data were represented as mean ± SD. ^**^P < 0.01. Each dot indicated a biological replicate, n = 8

The angiogenesis in the ischemic gastrocnemius muscle was then assessed. The infiltration of new capillaries (CD31^+^/α-SMA^−^) (Fig. 8A, B) and small arterioles (Fig. 8A, C) (CD31^+^/α-SMA^+^, defined as endothelial cells surrounded by α-SMA^+^ smooth muscle cells (Yeghiazarians et al. 2009) was significantly increased (Fig. 8A–C). An obvious increase in VEGFA content in the ischemic gastrocnemius muscle of diabetic mice was validated (Fig. 8D). The activation of VEGFR2/PI3K/AKT induced by AURKA overexpression was also observed in vivo, as evidenced by increased the expression of VEGFR2, p-PI3K, and p-AKT under HG and ND conditions (Fig. 8E–H).

### AURKA overexpression rescues ischemia-induced oxidative stress in murine gastrocnemius muscle

**Fig. 9:**
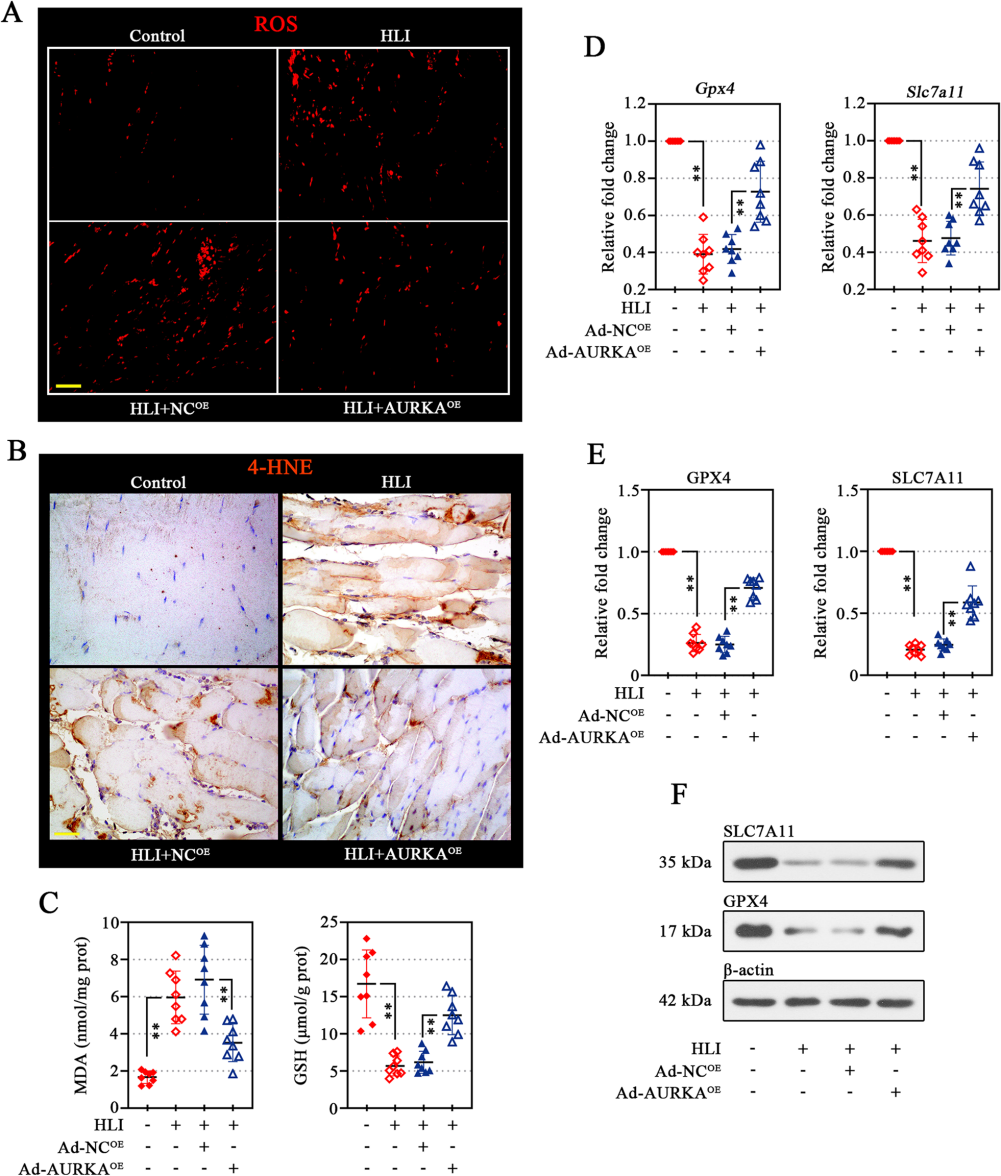
AURKA overexpression rescues ischemia-induced oxidative stress in murine gastrocnemius muscle. ROS production in the gastrocnemius muscle tissues was visualized by dihydroethidium (DHE) staining. Scale bar, 50 μm (**A**). 4-HNE expression in the gastrocnemius muscle tissues as shown by immunohistochemical (IHC). Scale bar, 50 μm (**B**). MDA and GSH content was measured using commercial assay kit (**C**). Besides, the mRNA (**D**) and protein (**E**, **F**) expression of GPX4 and SLC7A11 was detected. Data were represented as mean ± SD. ^**^P < 0.01. Each dot indicated a biological replicate, n = 8

Under ischemic diabetes, the role of overexpressed-AURKA on oxidative stress and following lipid peroxidation was verified. Ischemia-induced ROS production in gastrocnemius muscle was significantly restrained by AURKA overexpression (Fig. 9A), along with the promotion of GSH synthesis (Fig. 9C, right). In ischemic gastrocnemius muscle, determination of the levels of MDA (Fig. 9C, left) and 4-HNE (Fig. 9B) (lipid peroxidation by-products) produced by oxidative stress confirmed the inhibitory effect of AURKA overexpression on lipid peroxidation. As expected, AURKA overexpression promoted the expression of GPX4 and SLC7A11 (coordinating lipid peroxidation) induced by ischemic diabetes in the gastrocnemius muscle (Fig. 9D–F).

## Discussion

In our study, the in vitro and in vivo findings revealed the essential role of AURKA in regulating the angiogenic potential of microvascular endothelial cells under physiological and pathological conditions and the angiogenesis under post-diabetic ischemia conditions. To our knowledge, our study was the main report demonstrating the association and mechanism between AURKA and angiogenesis in diabetes-related CLI.

Vascular occlusion is the key pathological feature of ischemic disease, which is exacerbated by a deficit in angiogenesis. Endogenous new vessel formation and microvascular angiogenesis are the vital mechanisms in response to severe ischemia in the hind limb (Isner and Asahara 1999), and these mechanisms are mediated by the proliferation and invasion of endothelial cell and tube formation (Potente et al. 2011). These phenomena were compatible with a role of AURKA observed in our in vitro model, similar to that we had observed in vivo. In atherosclerosis, the upregulated AURKA could promote the proliferation and migration of umbilical vein endothelial cells, which was similar to our results (Liu et al. 2019). Cell proliferation is tightly related to cell cycle. The role of AURKA in cell cycle regulation has been widely verified, which mainly functions in the phase of G2 (Marumoto et al. 2002) by up-regulating cell cycle regulation-related genes such as CDK1 and Cyclin B (Vo et al. 2017). Similar to what was reported by Chen (2019) and Van Horn et al. (2010), we observed that AURKA overexpression promoted the cell cycle progression by promoting G2/M arrest by promoting Cyclin B1 and CDK1 expression. These data indicated that AURKA might improve cells to pass the G2/M transition checkpoint to regulate the passage of cells through the cell cycle, thus promoting cell proliferation.

VEGFA is a potent angiogenic parameter given its stimulating effect on endothelial cell motility, permeability, and tube formation to create a microvascular network (Apte et al. 2019). The decreased VEGFA expression in our study was consistent with other published reports in diabetic limb ischemia (Barć et al. 2021), and AURKA overexpression recovered decreased VEGFA expression and tube formation ability of endothelial cells induced by diabetes and ischemia. In diabetic-related ischemia, VEGFA signaling impairment is the key to this, which is characterized by decreased expression and sensitivity of endothelial cells to VEGFA (Rivard et al. 1999). VEGFA-induced angiogenesis is mediated by the activation of VEGFA receptor 1 (VEGFR1) and VEGFR2, and VEGFR2 is the primary contributor to its angiogenic effects (Webber et al. 2011). Ki et al. (2020) have reported that the activation of AURKA could induce the expression of VEGFA and VEGFR2 in endothelial cells under hypoxic conditions to promote endothelial dynamics, which consistent with the results observed in our research. The overexpression of VEGFR2 not only promotes angiogenesis but also activates other signaling pathways, inter alia including the PI3K/AKT pathway, which is responsible for cell survival and proliferation (Simons et al. 2016). In diabetic limb ischemia, the inhibition of PI3K/AKT pathway has been unveiled (Khaled et al. 2023). Further, similar to our results, AURKA was reported to activate the PI3K/AKT pathway to increase VEGFA expression, implying the potential role of AURKA in modulating multiple aspects of VEGFA expression in diabetic limb ischemia.

In diabetic ischemia, the endothelial cell is the primary site of hyperglycemic damage, where d-glucose tends to accumulate to decrease its sensitivity to VEGFA (Caporali et al. 2015). In cancer (Sun et al. 2020) and diabetes (Yin et al. 2020), AURKA has been demonstrated to function in glucose metabolism, suggesting that AURKA might promote the high glucose metabolism in microvascular endothelial cells to relieve VEGFA resistance. Thus, we proposed that AURKA might stimulate microvascular endothelial cells to form a vascular network via two distinct mechanisms: induction of VEGFA expression in diabetes-related limb ischemia and increased sensitivity of endothelial cells to VEGFA. The pro-angiogenic effect of ARUAK via VEGFA was further validated in Matrigel plugs. The increased functional capillaries (CD31 staining) containing erythrocytes (confirmed by hemoglobin content) suggested that AURKA was required to promote the migration of endothelial cells to induce VEGF-induced neovascularization. However, it is not yet known the underlying mechanism of how AURKA regulates angiogenesis via VEGFA.

The in vivo function of AURKA in post-ischemic angiogenesis related to diabetes was verified in our murine model of diabetic limb ischemia, and the results were consistent with in vitro experiments. The sample in our analysis is from the gastrocnemius muscle, where angiogenesis is most prevalent (Annex 2013). Our blood flow imaging analysis clearly showed that AURKA overexpression favors faster and more extensive reperfusion of the ischemia limb. The increased capillary density proved the critical role of AURKA in post-ischemic angiogenesis, with capillaries with lumens abundantly present. The adequate perfusion of capillary predominantly contributes to organ perfusion induction (Aref and Vries 2019). Of note, the elevation of small arteries in ischemic muscle might also favor the arteriogenic remodeling of collateral arteries in ischemic limb, leading to rapid blood flow recovery. These implied that AURKA overexpression permitted the recovery in perfusion in diabetic limb ischemia through both greater capillary sprouting and greater arteriogenesis, which might be mediated by VEGFA. The new formation of capillary tubes and the enhancement of vascular perfusion were reported to be primarily mediated by VEGF/VEGFR2 (Ferrara 2004).

Research has reported that the enhanced healing and/or regenerative capacity after ischemia is attributed to increased capillary and arterial vascular density in the ischemic limb, and the potential role of AURKA in wound repair has been hinted in diabetes (Yin et al. 2020). The healing of wound and functional restoration of tissue are important determinants of lower extremity function recovery. In our study, the increased VEGFA expression in gastrocnemius muscle was similar to Wagatsuma et al. (2007). VEGFA-stimulated angiogenesis contributes to the functional and morphological recovery of ischemia tissues in terms of the supplement of oxygen and nutrients to the damaged tissue (Deveci et al. 2002) and is considered as indispensable for new fiber formation after muscle damage. The data obtained from Desmin expression and morphological analysis revealed that AURKA was instrumental in new muscle regeneration and fiber formation. The damaged muscle function relies on an equilibrium between regeneration and fibrosis. Thus, AURKA might be considered as a contributor to protect against diabetes-related limb ischemia for its critical role in promoting angiogenesis and salvaging ischemic tissues.

Increased oxidative stress is the characteristic feature of endothelial cell dysfunction and vascular pathology both in human and animals (Huang 2009), and it is also induced by unregulated high glucose levels (Whiting et al. 2008). Diabetes and ischemia-induced the accumulation of ROS mediated endothelial cell death and decreased anti-oxidant enzyme level which is required for GSH synthesis (RamPravinKumar et al. 2021), and the above phenomena were confirmed in our in vivo and in vitro studies. The depletion of AURKA could promote ROS production thereby inducing cell apoptosis (Wang et al. 2016), suggesting its potential anti-oxidant effect in diabetic limb ischemia. In addition, lipid peroxidation induced by ROS also contributes to damage and cell death. In the experimental diabetic model of limb ischemia, the increased lipid peroxidation markers MDA and 4-HNE were observed (Edrees et al. 2002; Köksoy et al. 2007), and these were consistent with our data. In addition, the decreased ALOX4 and ACSL4 expression induced by AURKA overexpression. Both important enzymes to catalyze lipid peroxidation, was also confirmed its potential effect on lipid peroxidation.

Lipid peroxidation is the driving force for ferroptosis characterized by lipid-ROS accumulation. Nevertheless, reports on ferroptosis involvement in diabetic limb ischemia even in limb ischemia were not available. These results suggested that scavenging of excessive ROS to alleviate oxidative stress by AURKA might be another approach to help the survival and function of endothelial cells for angiogenesis. The inhibition of cysteine uptake results in reduced GSH levels and then suppressed Xc system (SLC7A11) and the following GPX4 expression, ultimately the induction of ferropotosis (Cao and Dixon 2016; Dixon et al. 2012). In type 2 diabetes, due to the limitation on cysteine and glycine availability, the deficiency of GSH is regarded as an important factor for elevated oxidative stress (Sekhar et al. 2011). These observations prompted us to test for possible ferropotosis involvement and AURKA function in diabetic limb ischemia. The decreased lipid ROS accumulation and GSH content as well as the expression of ferroptosis related genes (GPX4, SLC7A11, ALOX5, and ACSL4) implied the possible involvement of ferroptosis. Along with a similar result reported recently the data suggested that ARUKA might be involved in the negative regulation of ferroptosis in endothelial cells under diabetic and ischemic conditions. But, it should be noted that theses parameters do not accurate reflect the involvement of ferroptosis as it gold examination way is the shrunken mitochondria by transmission electron microscopy. Due to limited experimental conditions ad current, the data of the paper were predicted outcomes only.

Although our study gains the favorable results, however, some limitations still existed in our current study. Firstly, the samples from the dataset used to screen out the AURKA were the end phase of CLI patients; while, the sample from our experimental ischemia mouse was the acute ischemic insult. The involvement of AURKA might not be straightforward between two situations. Secondly, there is a limitation for LDPI method to monitor reperfusion changes within deeper muscular structure. Thirdly, our studies support the role of AURKA as a critical angiogenic factor in diabetes-related limb ischemia, and preliminary explored the possible involvement of VEGFR2/PI3K/AKT pathway. But, elucidating the specific mechanisms needs further research by using specific inhibitors for subsequent investigation. Fourthly, the direct involvement of ferroptosis and the relationship between ferroptosis and AURKA in diabetic limb ischemia had not been unambiguously demonstrated. Even though changes of biomarker for ferroptosis were observed. Furthermore, in our in vivo model, we could not exclude that adenovirus was used as a delivery vector, bringing about the triggering of significant immune responses and non-specific transfection to corresponding angiogenic phenomena. This also drove us to think about further development of the use of endothelial-specific knockdown/overexpression plasmids or mice to further investigate function and mechanism.

## Conclusions

**Fig. 10:**
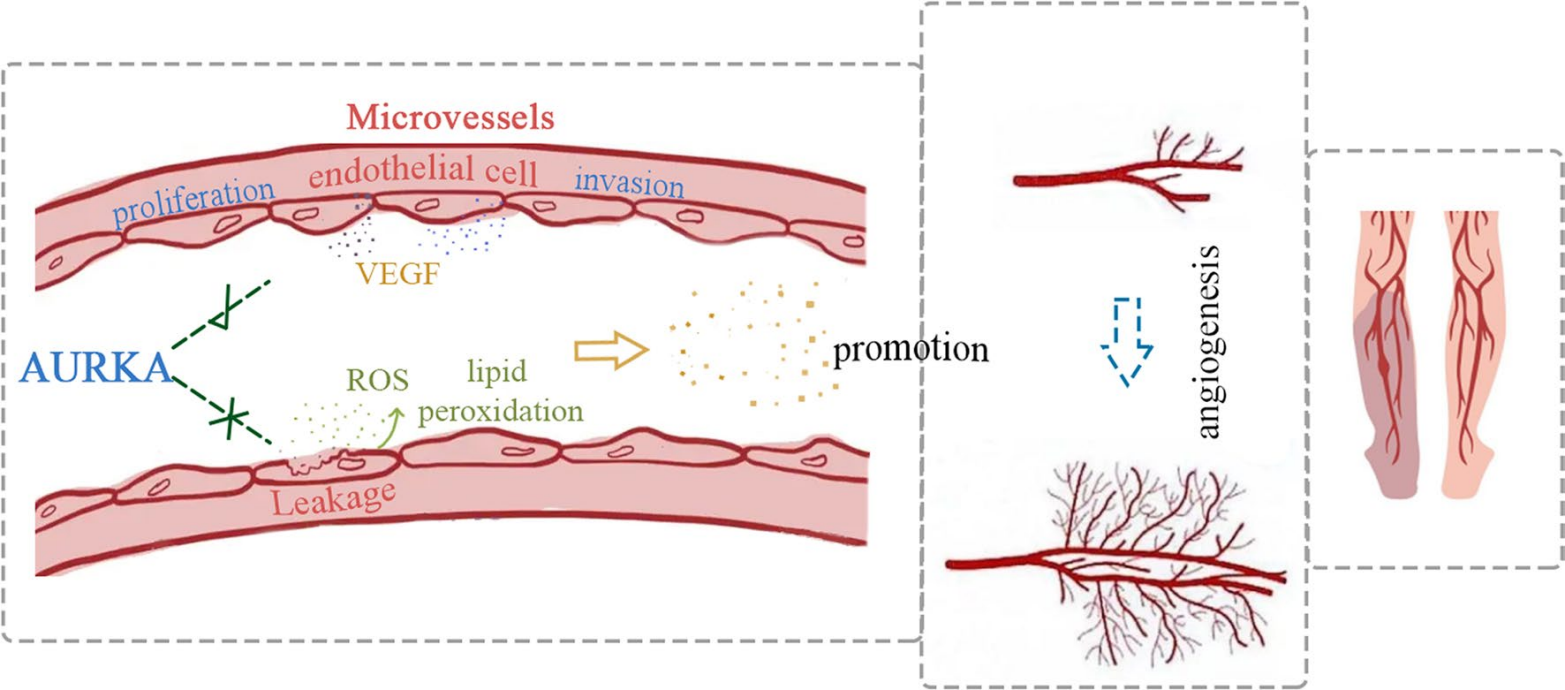
A diagram illustrating the regulation mechanism of AURKA in diabetic limb ischemia. AURKA overexpression under diabetic limb ischemic condition promotes the biologic role in angiogenic endothelial cell behavior and obstructed oxidative stress to boost angiogenesis

In conclusion, our research improved that AURKA plays an important regulatory role in post-ischemic angiogenesis related to diabetes via the promotion of endothelial proliferation and oxidative stress response. AURKA might promote endothelial cell proliferation by cycle modulating and modulate angiogenesis by targeting VEGFA through the VEGFR2/PI3K/AKT pathway. These provide a potential reference for diabetic limb ischemia management (Fig. 10).

## Data Availability

The datasets used and/or analyzed during the current study are available from the corresponding author on reasonable request.

## Abbreviations

CLI: Critical Limb Ischemia
PAD: Peripheral artery disease
HG: High glucose condition
ND: Nutrition deprivation
AURKA: Aurora Kinase A
VEGFA: Vascular endothelial growth factor A
STZ: Streptozotocin
HMEC-1: Human microvascular endothelial cells
FBS: Fetal bovine serum
FITC: Fluorescein isothiocyanate
PBS: Phosphate buffer saline
BrdU: Bromodeoxyuridine
MTT: 3-(4,5-Dimethylthiazol-2-yl)-2,5-diphenyltetrazolium bromide
DCFH-DA: 2′,7′-Dichlorofluorescein diacetate
BSA: Bovine serum albumin
MDA: Malondialdehyde
GSH: Glutathione
SLC7A11: Solute Carrier Family 7 Member 11
GPX4: Glutathione Peroxidase 4
ACSL4: Acyl-CoA Synthetase Long Chain Family Member 4
ALOX5: Arachidonate 5-Lipoxygenase
